# Identification and Validation of Angiogenesis-Related Gene Expression for Predicting Prognosis in Patients With Ovarian Cancer

**DOI:** 10.3389/fonc.2021.783666

**Published:** 2022-01-03

**Authors:** Yue Wang, Bao Xuan Li, Xiang Li

**Affiliations:** Department of Obstetrics and Gynaecology, Shengjing Hospital of China Medical University, Shenyang, China

**Keywords:** angiogenesis, ovarian cancer, prognosis, biomarkers, tumor immune microenvironment

## Abstract

Ovarian cancer (OC) is a highly heterogeneous disease with different cellular origins reported; thus, precise prognostic strategies and effective new therapies are urgently needed for patients with OC. A growing number of studies have shown that most malignancies have intensive angiogenesis and rapid growth. Therefore, angiogenesis plays an important role in the development of tumor metastasis. However, the prognostic value of angiogenesis-related genes (ARGs) in OC remains to be further elucidated. In this study, the expression data and corresponding clinical data from patients with OC and normal control samples were downloaded with UCSC XENA. A total of 1,960 differentially expressed ARGs were screened and functionally annotated through Gene Ontology (GO) terms and Kyoto Encyclopedia of Genes and Genomes (KEGG) pathways. Univariate Cox regression analysis was performed to identify ARGs associated with prognosis. New ARGs signatures (including *ESM1*, *CXCL13*, *TPCN2*, *PTPRD*, *FOXO1*, and *ELK3*) were constructed for the prediction of overall survival (OS) in OC based on the least absolute shrinkage and selection operator (LASSO) and multivariate Cox regression analysis. Patients were divided based on their median risk score. In the The Cancer Genome Atlas (TCGA) training dataset, the survival analysis showed that overall survival was lower in the high-risk group than that in the low-risk group (p < 0.0001). The International Cancer Genome Consortium (ICGC) database was used for validation, and the receiver operating characteristic (ROC) curves showed good performance. Univariate and multivariate Cox analyses were conducted to identify independent predictors of OS. The nomogram, including the risk score, age, stage, grade, and position, can not only show good predictive ability but also can explore the correlation analysis based on ARGs for immunogenicity, immune components, and immune phenotypes with risk score. Risk scores were correlated strongly with the type of immune infiltration. Furthermore, homologous recombination defect (HRD), NtAIscore, LOH score, LSTm score, stemness index (mRNAsi), and stromal cells were significantly correlated with risk score. The present study suggests that the novel signature constructed from six ARGs may serve as effective prognostic biomarkers for OC and contribute to clinical decision making and personalized prognostic monitoring of OC.

## Introduction

Ovarian cancer (OC) is a global problem, ranking eighth in mortality and morbidity among women. According to the data from the 2020 GLOBOCAN, an estimated 313,959 people worldwide were diagnosed with OC in 2020, accounting for 3.4% (431,288 cases) of all new cases of cancer and 4.7% (207,252 deaths) of all new deaths due to cancer (1). Moreover, due to its insidious clinical presentation and no effective screening method in the early stage, most cases (almost 75%) are diagnosed at its late stage, resulting in a poor 5-year survival rate (2). The current standard front-line treatment, including cytoreductive surgery and a combination chemotherapy, such as platinum and paclitaxel, has been performed on patients with OC, with a 5-year survival rate of OC with stage III–IV <20% (3). Therefore, to explore and establish a reliable prognostic model of OC is of great significance to guide more appropriate clinical treatment and improve the prognosis of OC.

Angiogenesis factors (AFs) are essential for tumorigenesis because of their indispensable induction in providing oxygen and delivering nutrients and metastatic conduits (4). In 1971, Folkman first proposed that the development of tumors depends on angiogenesis (5). In recent decades, numerous studies have found that AFs can induce angiogenesis, increasing the aggressiveness of tumors by promoting tumor-associated neovascularization, which are essential for the development and metastasis of tumors (6, 7). Some angiogenesis inhibitors have been recommended for treatment and approved for many cancers (8). Many studies have demonstrated that some AFs play an important role in the development of OC, such as vascular endothelial growth factor (VEGF), hypoxia-inducible factor 1 α (HIF-1α), phosphatase and tensin homolog (PTEN), and miR-205 (9–11). Thus, the expression of angiogenesis-related genes (ARGs) may be a potential target for OC.

In this study, we established the ARG risk models based on ARGs in the NCBI-Gene and MSigDB databases to predict the overall survival (OS) in OC in the Xena-OC dataset and validate it in the ICGC-OV-AU dataset. Then, we further revealed the relationship between high- and low-risk subgroups and immune infiltration with biofunctional prognosis. Overall, our data suggest that ARGs play a key role in the pathogenesis of OC, which are potential therapeutic targets and prognostic markers for OC, providing a more effective approach to prevent tumor progression and treat cancer metastasis.

## Materials and Methods

### Data Acquisition

The data used in this study, including the fragments per kilobase of transcript per million mapped reads (FPKM) standardized sequencing dataset and the corresponding clinical data (patients, age, and other clinical information of patients) of OC samples and non-OC samples, were all retrieved from open-source databases, such as the UCSC Xena (https://xenabrowser.net/datapages/), TCGA dataset (http://www.tcga.org), and GTEx database (https://gtexportal.org/). All the data were removed for batch effects with the “sva” package in R (https://www.rproject.org/). At last, 88 normal and 354 tumor samples were obtained. Another OC dataset from the International Cancer Genome Consortium (ICGC) data portal (https://icgc.org/) was obtained as the external verification database, including 111 tumor samples. The list of 1,960 ARGs retrieved from NCBI-Gene and MSigDB databases with the keyword “angiogenesis.”

### Identification of Differentially Expressed ARGs

The “limma” package was adopted to identify the difference between ARGs in training cohort, with the cutoff criteria of |log2 fold change (FC)| >1 and an adjusted p < 0.05 [false discovery rate (FDR)] as the criteria for ARGs identification. Heatmap of differential ARGs was expressed based on R package “pheatmap,” and Volcano plots were drawn with R package “ggpubr.”

### GO and KEGG Functional Enrichment Analysis of Different ARGs

To reveal the potential pathways and biological functions of ARGs, Kyoto Encyclopedia of Genes and Genomes (KEGG, http://www.kegg.jp/) analysis and Gene Ontology (GO, http://www.geneontology.org) analysis with the “clusterProfiler” package in R software were also utilized in this study. The top 20 GO terms were visualized based on R package “enrichplot,” and KEGG chord diagram were drawn by R package “GOplot.”

### Identification of Signatures of Survival-Related ARGs

The prognostic ARGs were identified to determine statistical significant correlation with OS of patients with OC through the univariate Cox regression analysis by the “survival” package, with p < 0.05 as the threshold. The subsequent survival curve was plotted. The expression levels of ARGs were divided into high and low groups by median to demonstrate the prognostic differences between the subgroups. A box plot was plotted by R package “ggpubr” to assess the comparability of groups.

### Construction and Validation of a Risk Model Based on ARGs

After the univariate Cox regression analysis and forest plots were established with the “forestplot” package, the prognostic ARGs associated with OC by least absolute shrinkage and selection operator (LASSO) regression and multivariate COX regression were further screened out. First, LASSO regression analysis was conducted to establish a prognostic multigene signature in the training set with the glmnet package. The dependent variable in the LASSO regression analysis was overall survival and status of patients in the TCGA cohort, and the independent variable in the LASSO regression analysis was standardized expression matrix of candidate prognostic ARGs. Then, a risk model was constructed through multivariate Cox regression analysis to distinguish the significant prognostic ARGs, with the final prognostic ARGs of patients with OC on the basis of the linear combination of regression coefficient (β) obtained from the LASSO-Cox regression model and their gene expression levels.

### Building and Evaluation of the Prognostic Signatures of ARGs

First, according to the risk-scoring formula, ARGs were divided into low- and high-risk groups with the median risk score as the cutoff point. The survival time of patients was demonstrated by plotting scatter plot survival with R package “ggplot2.” Subsequently, the displayed heatmaps showed the expression profiles of the prognosis AFGs based on R package “ComplexHeatmap,” with survival curves for high- and low-risk groups plotted by R packages “survminer” and “survival.” Finally, the model was validated by receiver operating characteristic (ROC) curves plotted with R package “pROC” and “survivalROC,” and area under curve (AUC) of multiple time points was calculated to evaluate the recognition effects.

Then, the prognostic signature was verified with the same coefficients and cutoff value in the external dataset, ICGC-OV-AU; the prognostic model was presented as a risk map at the same time, covering the expression levels of the contained genes, the distribution of risk score, and the survival status of individuals.

Furthermore, the independent prognostic factor of the prognostic signature was explored through univariate and multivariate Cox analyses.

Finally, the nomograph was constructed with R packages “rms,” which assessed the survival of patients with risk score and clinical characteristics, and the subsequent ROC survival curve was plotted based on R packages “urvivalROC.”

### Relationship Between Risk Model, Immunity, and Tumor Microenvironment

First, the correlation between ARG risk score and each indicator, including mutational load, homologous recombination defect (HRD), neoantigen load and chromosomal instability, and stemness index (mRNAsi), was analyzed with R package “ggpubr.” Mutational load was calculated using the R package “maftools.” The data of homologous recombination defect (HRD) and neoantigen load and chromosomal instability were obtained from the appendix of the article (PMID: 29617664). The stemness index (mRNAsi) was assessed based on expression profiles using the assessment algorithm from Tathiane M.Malta. Then, the proportion of 22 tumor-infiltrating immune cells (TIICs) in the Xene-OC samples was analyzed *via* the CIBERSORT algorithm (https://cibersort.stanford.edu/), and the differential expression was conducted in high- and low-risk groups by R package “ggpubr”. Moreover, immune scores of high- and low-risk groups were calculated with the package “estimate”, and plot histograms of differences in immune scores, stromal scores, and tumor purity of high- and low-risk groups with R package “ggpubr”.

### Statistical Analysis

All the statistical analyses were performed with R software (version 4.0.3, http://www.r-project.org), with the visualization on the results. Kaplan–Meier method was applied to calculate the cumulative survival time and the log-rank test from the survival package to analyze the differences in survival curves. Cox proportional risk regression models were applied for univariate and multivariate analyses. p < 0.05 means the difference is statistically significant.

## Results

### Identification of ARGs

**Figure 1:**
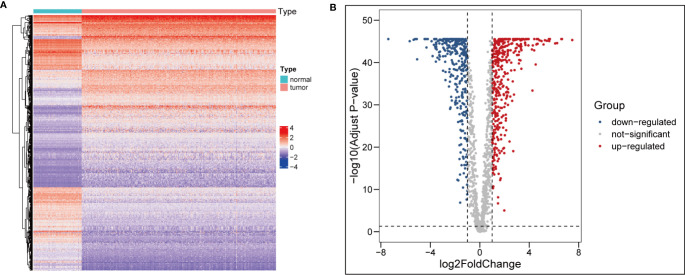
Identification of differentially expressed ARGs. **(A)** heatmap of differential ARGs in ovarian cancer. **(B)** Volcano map of differential ARGs.

After comparing the different expression of ARGs between OC tissues (n = 354) and adjacent normal tissues (n = 88), 1,960 ARGs (|log2FC| > 1, adj.pvalue < 0.05) remained, including 330 upregulated and 397 downregulated genes (**Figures 1A, B** **)**.

### Functional and Pathway Enrichment Analysis of Different ARGs

**Figure 2:**
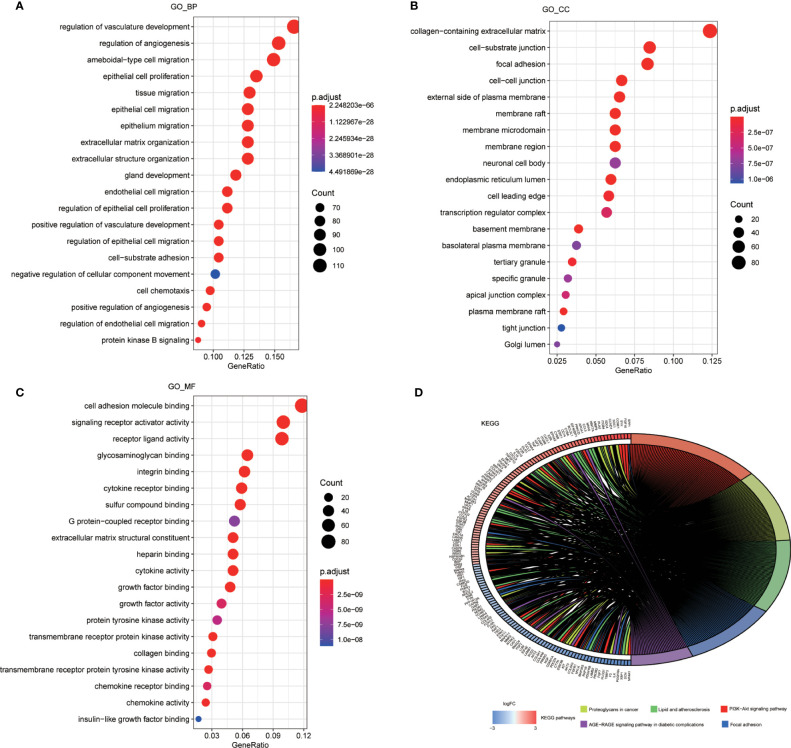
Enrichment analysis of ARGs. **(A)** Top 20 most significant biological processes in GO analysis. **(B)** Top 20 most significant cellular components in GO analysis. **(C)** Top 20 most significant molecular function in GO analysis. **(D)** Top 20 most significant KEGG pathways.

GO and KEGG pathway enrichment analysis were performed to reveal biological processes, cellular components, and molecular functions of ARGs, with p < 0.05 and enrichment >2.0 as the cutoff criteria. The top 20 GO and KEGG pathway enrichment analyses were visualized in the bubble diagram ways (**Figure 2**). The top 3 GO_BPs (biological processes) were vascular development regulation (GO:1901342), angiogenesis regulation (GO:0045765), and amoeboidal-like cell migration (GO:0001667) (**Figure 2A**). The top 3 GO_CCs (cellular components) were collagen-containing extracellular matrix (GO:0062023), cell–substrate junction (GO:0030055), and focal adhesion (GO:0005925) (**Figure 2B**). The top 3 GO_MFs (molecular functions) were cell adhesion molecule binding (GO:0050839) (**Figure 2B**), signaling receptor activator activity (GO:0030546), and receptor ligand activity (GO:0048018) (**Figure 2C**).

According to the KEGG enrichment analysis, PI3K-Akt signaling pathway played a significant role in patients with OC. In addition to proteoglycans in cancer, lipid, and atherosclerosis, focal adhesion and age-rage signaling pathway in diabetic complications were also suggested as important pathways in tumor progression (**Figure 2D**).

### Identification of Survival-Related ARGs

**Figure 3:**
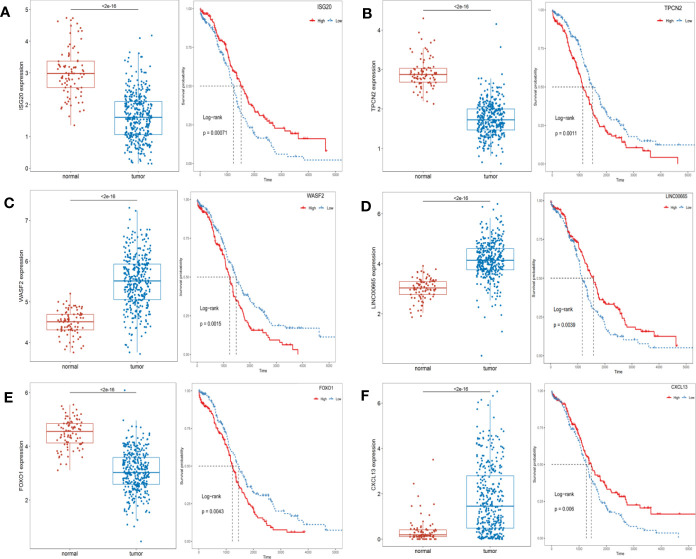
The expression levels of prognosis-related top 6 ARGs between the normal and OC patients and Kaplan-Meier curves in the high- and low-risk groups. **(A)** ISG20; **(B)** TPCN2; **(C)** WASF2; **(D)** LIINC00665; **(E)** FOXO1; **(F)** CXCL13.

Univariate Cox regression analysis showed that a total of 59 differential expressed ARGs significantly related with the survival were identified (*p* < 0.05), which were then divided into high and low groups by median, and six genes with the smallest P were selected to demonstrate the prognostic differences between the groups (**Figure 3**). As shown in **Figure 3**, *ISG20*, *TPCN2*, and *FOXO1* were lowly expressed in OC patients, and their low expression predicted a poor prognosis, while *WASF2*, *LIINC00665*, and *CXCL13* were highly expressed in OC patients, and their high expression also predicted a poor prognosis.

### Construction and Validation of a Risk Model Based on Six ARGs

In the training set from Xena-OC, a total of 59 ARGs were screened out as prognosis-related genes through the univariate Cox regression analysis (*p* < 0.05). The forest map showed that most of the top 20 ARGs were risk genes (**Supplementary Figure S1A**), which were then further reduced in LASSO Cox regression method (**Supplementary Figure S1B**). Finally, the six ARGs were reserved based on the multivariate COX regression method (**Supplementary Figure S1C**).

Risk score = −0.23767*(expression level of *ESM1*)

+(−0.20947)*(expression level of *CXCL13*)

+0.35813*(expression level of *TPCN2*)

+0.30918*(expression level of *PTPRD*)

+0.18439*(expression level of *FOXO1*)

+0.24544*(expression level of *ELK3*)

### Estimation and Validation on the Signatures of ARGs

**Figure 4:**
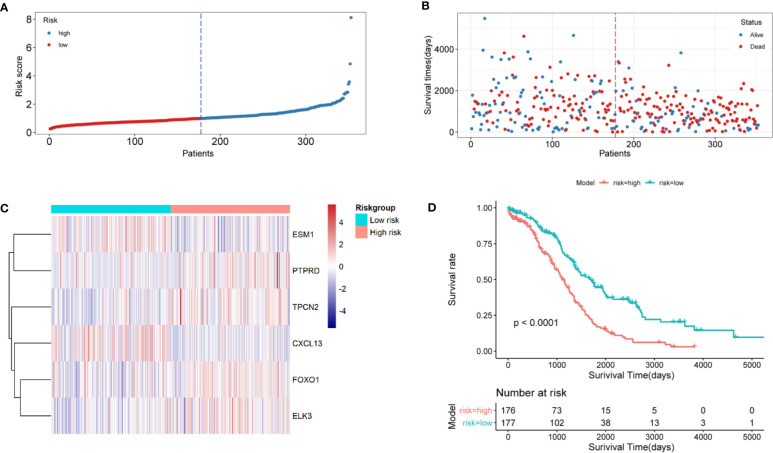
Validation of ARG signature in the Xena-OC dataset. **(A)** Risk score distribution and high- and low-risk groups; **(B)** survival statuses in high- and low-risk groups; **(C)** heatmap of six AF-related genes in high- and low-risk groups; **(D)** time-dependent ROC curves in high- and low-risk groups.

In the Xena-OC dataset, the risk score was calculated for each patient, which were then divided into high- and low-risk groups by median (**Figure 4A**). As can be seen from the figure, there are significant differences in survival time, differential expression levels of AF-related gene signatures, and survival rate (**Supplementary Figure 2**). The scatter plot shows that each group has different survival status, with blue dots indicating survival and red dots indicating death (**Figure 4B**), revealing that the number of patients dying gradually decreased as the risk score decreased throughout the follow-up. We can see that there is a slight difference in the extreme region of the risk score. Heatmaps showed the distribution of six AF-related genes (**Figure 4C**). The survival analysis revealed that the OS rate of high-risk group was significantly shorter than that of low-risk group (p < 0.001), which shows that the risk score and survival time are significantly correlated (**Figure 4D**).

The conclusions of this model were verified in the external ICGC dataset. In the ICGC-OV-AU dataset, the same cutoff values were calculated for the risk score of each patient. Similarly, the mortality rate was higher in the high-risk group, while the majority of patients in the low-risk group remained alive during the follow-up (**Supplementary Figure S2B**). Heatmaps revealed the distribution of the six AF-related genes (**Supplementary Figure S2C**). In the ICGC-OV-AU dataset, a total of 81 patients had survival data. According to the survival analysis, patients in the high-risk group had significantly lower OS than those in the low-risk group (**Supplementary Figure S2D**). Both the positive and negative groups consisted of OC patients who survived for 1, 3, and 5 years. The positive group comprised the patients whose predicted outcome was consistent with the actual outcome after model prediction, and the negative group comprised the patients whose predicted outcome was inconsistent with the actual outcome after model prediction. In this study, the accuracy and sensitivity of the model were judged by the time-dependent ROC curve with specificity as the horizontal axis and sensitivity as the vertical axis, in which the larger the AUC area, the higher the accuracy and sensitivity. As shown in the figure, ROC curves of OC samples showed that the 1-, 3-, and 5-year area under the curve (AUC) were 0.622, 0.662, and 0.705, respectively, in the model group, while those in the validation group were 0.703, 0.686, and 0.573, respectively, indicating that the performance of AF signature was very stable (**Supplementary Figure S3**).

### Univariate and Multivariate COX Regression Analyses of Risk Score

Univariate and multivariate Cox regression analyses were performed to determine whether risk score was an independent prognostic factor, which showed that the risk score was indeed an independent prognostic factor. In the training cohort, the univariate Cox regression analysis indicated that risk score was the only independent prognostic factor of OS in OC among age, stage, grade, disease status, lymphatic invasion, position, and risk score, and the multivariate Cox regression analysis revealed that the risk score was still the only independent prognostic factor for OS in OC after adjusting for age, stage, grade, disease status, lymphatic invasion, and position (**Supplementary Figure S4**).

### Construction of Nomogram

A visualization of column line graph was constructed according to risk score, age, stage, grade, and position (**Supplementary Figure S5A**). According to the standard score of each risk factor, the scores of each risk factor were obtained, with the sum of scores as the total score based on the above indicators, which could predict the 1-, 3-, and 5-year survival rates for each patient. The results of the multi-indicator ROC curve analysis combining the clinical characteristics showed that the AUCs of 1-, 3-, and 5-year survival risk score were higher than those of other clinical characteristics, which were 0.742, 0.686, and 0.658, respectively (**Supplementary Figure S5B**). Standard curves were adopted to assess the predictive ability of nomogram. As shown in **Supplementary Figures S5C–E**, the levels of 1-, 3-, and 5-year calibration curves overlapped well with the standard curve. Meanwhile, these results indicated that the risk score model has good predictive ability.

### Correlation of Risk Score With Immunity

**Figure 5:**
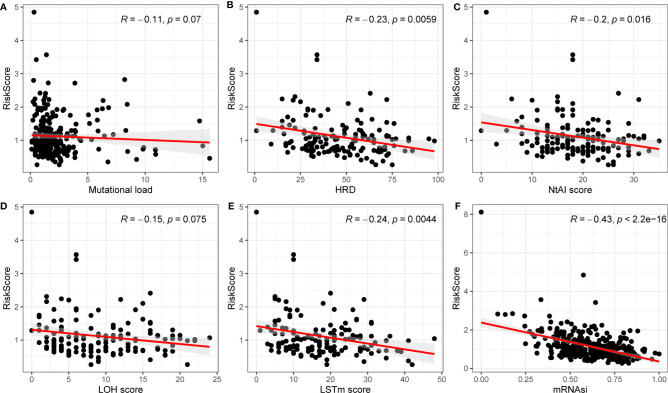
Correlations between mutation load, HRD, NtAI score, LOH score, LSTm score, mRNAsi and risk model. **(A)** Mutation load; **(B)** HRD; **(C)** NtAI score; **(D)** LOH score; **(E)** LSTm score; **(F)** mRNAi.

The risk score has been shown to be an independent prognostic factor for patients with OC, which were classified into high- and low-risk groups based on risk score. Then, the relationship between the high- and low-risk groups in mutational load, HRD, neoantigen load and chromosomal instability, mRNAsi, immune cells, and tumor microenvironment (TME) was investigated. As shown in **Figure 5**, HRD, NtAI score, LSTm score, and mRNAsi in the high- and low-risk groups was identified to be significantly varied. In the high-risk group, HRD, NtAI score, LSTm score, and mRNAsi obviously decreased. To investigate the potential mechanism between risk score and OS in patients with OC, multiple immunospectrum-related analyses were performed. The stromal scores and ESTIMATE scores differed significantly between the high- and low-risk groups.

**Figure 6:**
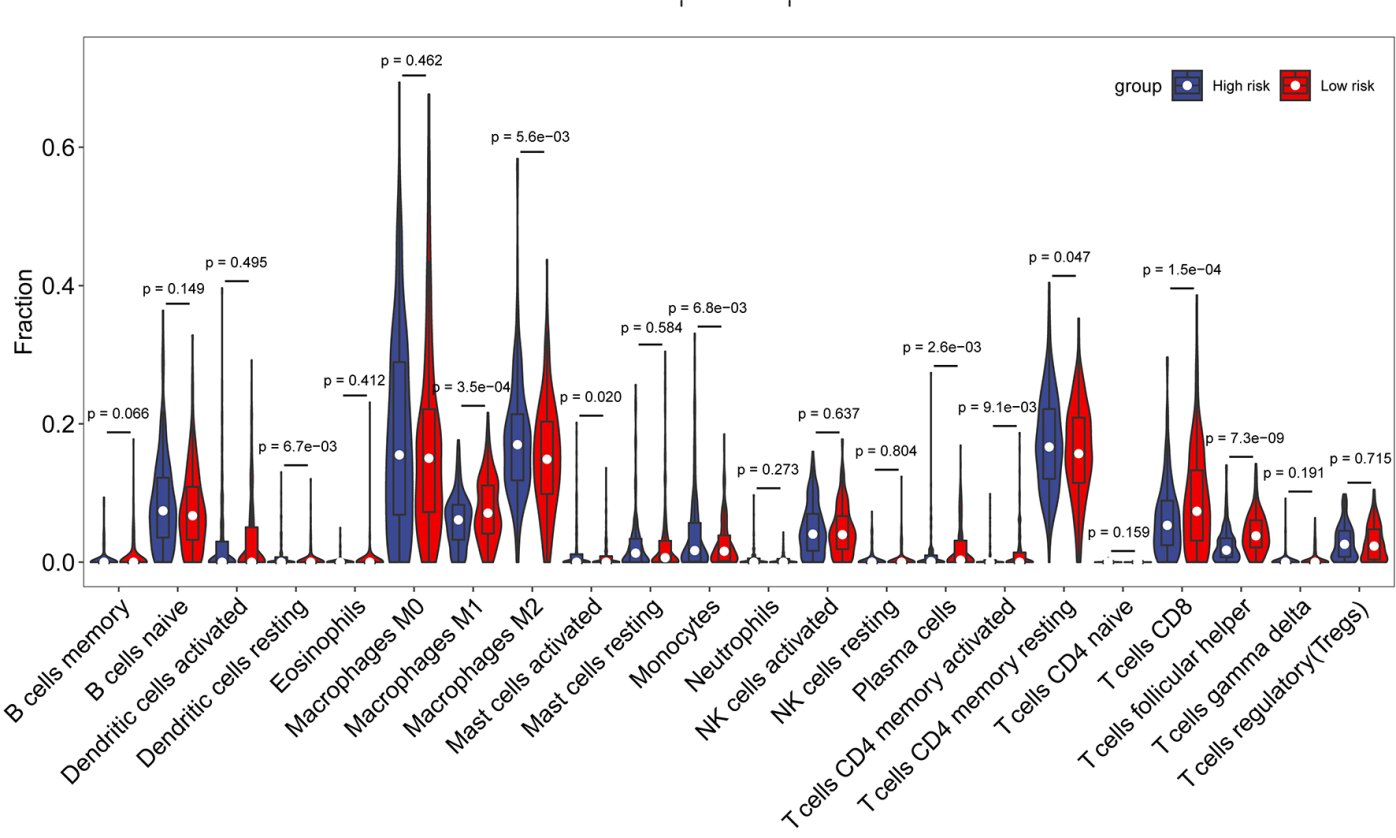
Comparison of immune cell composition between the high- and low-risk groups.

Then, R package “CIBERSORT” was applied to analyze the proportion of 22 immune cell types infiltrating each sample in the high- and low-risk groups, where samples with p < 0.05 were excluded to ensure the accuracy. As shown in **Figure 6**, there were some differences in the comparison of immune cells between the two groups of samples. Macrophages M1, plasma cells, CD8 T cells, and T follicular helper cells were higher in the low-risk group than those in the high-risk group, while macrophages M2, monocytes, and memory CD4 T cells were higher in the high-risk group than those in the low-risk group. The differences in immune cells between the low- and high-risk groups may be related to disease progression and tumor resistance to multiple treatments.

### Relationships Between Risk Model and Tumor Microenvironment

TME refers to the internal and external environment in which tumor occurs, grows, and metastasizes along with tumor cells, including not only tumor cells but also immune cells, stromal cells, etc.

The TME of the entire cohort was analyzed by an “estimate” package to generate the estimated, stromal, and immune cell scores. The estimated score, the sum of stromal cells and immune cell score, demonstrates the abundance of cancer cell components. Higher scores mean higher component frequency within the sample. It was found that comparing with the high-risk group, the low-risk group had significantly higher stromal score, slightly lower immune cell score, and higher estimated score. However, the difference between the two groups was not statistically significant (**Supplementary Figure S6**).

## Discussion

OC is one of the most serious malignancies in the female reproductive system worldwide and is often diagnosed at an advanced stage, with the rising incidence in recent years, making it a huge public health challenge worldwide. According to the International Federation of Gynecology and Obstetrics (FIGO) staging system, the treatments for patients with OC usually include tumor-reducing surgery and adjuvant or neoadjuvant chemotherapy, with conventional treatment options showing substandard efficacy, high recurrence rates, and chemoresistance, which are the main reasons for the low 5-year survival rate of OC (12). As a result, searching for reliable tumor markers and exploring accurate prognostic strategies and effective new therapies are significant for the treatment and prognosis of OC. Angiogenesis is the process of generating new capillaries from pre-existing blood vessels, such as capillaries and post-capillary microvenules, regulated by angiogenic and anti-angiogenic factors. Increasing studies have shown that angiogenesis can provide sufficient oxygen and nutrients to tumor cells with a key role in the biological behavior of tumor growth, invasion, and metastasis, and its inhibition will significantly prevent the development and spread of tumor tissues. Therefore, targeted therapy with angiogenesis inhibitors has been widely accepted as a clinical treatment strategy (8). However, numerous reports have indicated that the relative drugs have not yet shown significant benefits for patients (13), partly due to incomplete understanding about the mechanisms of tumor angiogenesis. As we know, angiogenesis is a complex process triggered by many genes, and many reports have shown that AFs are the potential prognostic gene is associated with OC. The integration of these AF-associated genes is often important for understanding the process of tumor invasion; however, the potential role of AF-associated gene signatures as an effective therapeutic strategy for OC remains unclear. Therefore, it is urgent to develop a predictive model for AF-related gene signatures, which can provide new insights for the individualized treatment of OC.

In the current study, TCGA OC data were combined with GTEx normal ovarian expression profile data to systematically analyze the expression of 1960 ARGs in OC tissues and their relationship with OS. In the current study, 727 differentially expressed ARGs substantially associated with survival by univariate COX regression analysis were obtained to explore the prognostic value of ARGs. Then, a prognostic model integrating six ARGs were constructed through the LASSO regression analysis and multifactorial COX regression methods, which was validated in the ICGC dataset. Patients were divided into high- and low-risk groups based on median risk score. KM survival analysis, ROC curves, and Cox regression analyses demonstrated that the high-risk group was significantly associated with shorter OS. To translate the ARGs risk model into further clinical practice, column line plots containing prognostic features and clinicopathological staging were drawn, and risk score, age, grading, and staging were constructed to predict 1-, 3-, and 5-year survival probabilities for patients with OC. Univariate and multivariate Cox regression analyses showed that risk score was an independent predictor for OS. The prognostic model was significantly better than the widely used clinical staging and FIGO staging, and its development could improve the management of patients with OC.

Many studies have shown that angiogenesis is involved in multiple signaling pathways, affecting the development and progression of OC. KEGG pathway analysis based on ARGs showed that PI3K-Akt signaling pathway, proteoglycan in cancer, lipids, and atherosclerosis, focal adhesion, and age-rage signaling pathway in diabetic complications played a significant role in patients with OC. A further study established a relationship with six ARGs (ESM1, CXCL13, TPCN2, PTPRD, FOXO1, and ELK3) characteristics associated with a new prognostic model. ESM1 is a human endothelial cell-specific molecule synthesized by tumor endothelial cells. Studies have shown that serum ESM1 levels are associated with survival time and tumor invasion in patients with cancer. It has also been proposed that ESM1 synthesized by tumor endothelial cells may represent a good marker of angiogenesis and may even be a potential therapeutic target for angiogenesis (14). CXCL13, a B-lymphocyte chemokine, is widely involved in the pathogenesis of inflammatory and autoimmune diseases and preferentially promotes B-lymphocyte migration and chemotaxis by stimulating calcium inward flow (15). CXCL13 has been shown to control the phenotype of cancer cells in various solid tumors and to affect the migration, invasion, and growth of cancer cells (16). TPCN2, two-pore segment channel 2, is a generally expressed, lysosome-targeting ion channel contributing to the termination of autophagy (17), which can affect autophagy progression and extracellular vesicle (EV) trafficking in cancer cells (18). The protein-encoded PTPRD, a member of the protein tyrosine phosphatase (PTP) family, is a signaling molecule that regulates a variety of cellular processes, including cell growth, differentiation, mitotic cycle, and oncogenic transformation. Studies have shown that PTPRD is frequently inactivated in various malignancies and lacks induction of tumor cell metastasis (19). FoxO is a transcription factor with a common DNA binding domain that exerts positive effects mainly on genes involved in cell cycle, apoptosis regulation, and drug resistance. Studies have shown that the decrease in FoxO1 expression is associated with the resistance to conventional drugs (e.g., cisplatin) and lower efficacy of drug combinations in OC cells (20). Other studies have also found that FOXO1 protein expression correlates with recurrence-free survival and OS in patients with OC. In addition, FOXO1 expression is correlated with age, FIGO stage, abdominal recurrence, and degree of differentiation (21). ELK3 (also known as Net, SAP-2, or ERP), a member of the ETS family, can be considered as a transcription factor that binds to specific DNA sequences rich in purine GGA core sequences and regulates the expression of a variety of genes, including proto-oncogenes (22).

Although the mechanisms of angiogenesis have been the focus of research in the past, the potential relationship between angiogenesis and tumor immunity remains unclear. As we know, under normal conditions, the immune system can recognize and remove tumor cells from the TME. However, tumor cells under the supervision of the immune system can develop multiple mechanisms to evade immune killing and thus survive at various stages of antitumor immune response, and therefore, the relationship between risk score and antigen presentation and immune escape in tumors was further investigated. First, to explore the effects of ARGs on immunogenicity in patients with OC, the potential determinants of tumor immunogenicity were first discussed, including mutational load, HRD, neoantigen load and chromosomal instability, and mRNAsi with risk score (23). Our findings revealed that mutation load, HRD, NtAIscore, LOH score, LSTm score, and mRNAsi were negatively relevant to risk score. Moreover, HRD, NtAIscore, LOH score, LSTm score, and mRNAsi were significantly correlated with risk score. As a whole, the tumor immunogenicity differed significantly between the high- and low-risk score groups. The low-risk score group had relatively low immunogenicity, and the high-risk score group had relatively high immunogenicity.

Subsequently, to explore the relationship between risk score and immune components, we investigated the role of risk score in the type of immune infiltration and immune score. First is the immune cells. There are many types of immune cells, and different types of immune cells in turn play different roles in anti-tumor and tumor immune escape processes, and tumor growth, invasion, and metastasis are invariably associated with immune cells. The next is stromal cells, which are also thought to play an important role in tumor growth, disease progression, and drug resistance. Studies have found that macrophages M1, plasma cells, CD8 T cells, and T follicular helper cells were higher in the low-risk group than in the high-risk group, while macrophages M2, monocytes, and memory CD4 T cells were higher in the high-risk group than in the low-risk group. We found that stromal score was significantly higher in the high-risk group than in the low-risk group. CD8+ T lymphocytes are the main anti-tumor effector cells (24). Studies have shown that infiltration of high levels of CD8 T cells may help tumor cell regression, leading to long-term remission of the disease (25). T follicle helper (Tfh) cells are protective in non-lymphoid tumors. High levels of Tfh cell infiltration are associated with increased overall tumor survival and decreased immunosuppression (26). These findings firmly suggest that this AFs’ signature affects prognosis by interfering with immune cell infiltration in OC.

According to the currently searchable literature, this is the first relatively comprehensive study to establish an ARGs prognostic model for patients with OC and develop prognostic-related line graphs. However, some limitations should also be noted in our study. First, our study is a retrospective study based on two public datasets with relatively small samples and limited data sources; thus, a larger sample size and more ethnic data are needed for validation. Second, although our study provides evidence that the six-gene signature is a significant predictor of OC survival, the underlying mechanisms between the signature genes and OC are not sufficiently clear, and we need to validate them through further translational research experiments such as cellular studies and animal experiments to check the predictive accuracy of the model and to discover the underlying mechanisms. Third, since this is a retrospective study with high interpatient variability, the results need prospective studies to verify their clinical applicability. Fourth, post-excision surgery residual lesion status is an important prognostic factor for OC, but insufficient information on excision status in our study led us to overlook the factor.

## Conclusions

In conclusion, AFs are critical for OC invasion and metastasis, which are associated with poor OS in patients with OC. In this study, the signatures of ARGs were identified for prognosis prediction in patients with OC, where a higher risk score indicates poorer prognosis. In addition, further elucidation of underlying mechanisms based on these genes can provide theoretical guidance for basic research, which may facilitate individualized treatment and clinical decision-making for patients with OC.

## Data Availability Statement

The datasets presented in this study can be found in online repositories. The names of the repository/repositories and accession number(s) can be found in the article/**Supplementary Material**.

## Author Contributions

YW and BL analyzed the gene expression data and wrote the paper. XL conceived the study and revised the content of this manuscript. All authors contributed to the article and approved the submitted version.

## Conflict of Interest

The authors declare that the research was conducted in the absence of any commercial or financial relationships that could be construed as a potential conflict of interest.

## Publisher’s Note

All claims expressed in this article are solely those of the authors and do not necessarily represent those of their affiliated organizations, or those of the publisher, the editors and the reviewers. Any product that may be evaluated in this article, or claim that may be made by its manufacturer, is not guaranteed or endorsed by the publisher.
