# Supplementary figures and images for: Identification and Validation of Angiogenesis-Related Gene Expression for Predicting Prognosis in Patients With Ovarian Cancer
Source: Front Oncol. 2022 Jan 3;11:783666. doi: 10.3389/fonc.2021.783666 (PMC8761815; doi:10.3389/fonc.2021.783666)

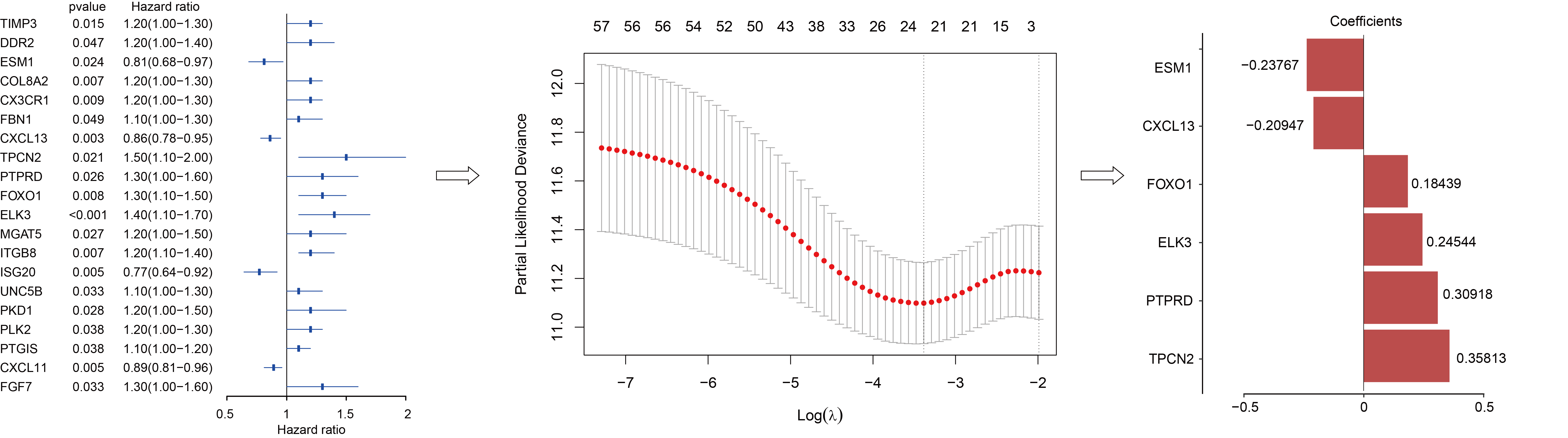

Supplement: Supplementary file 1 [file Image_1.png]

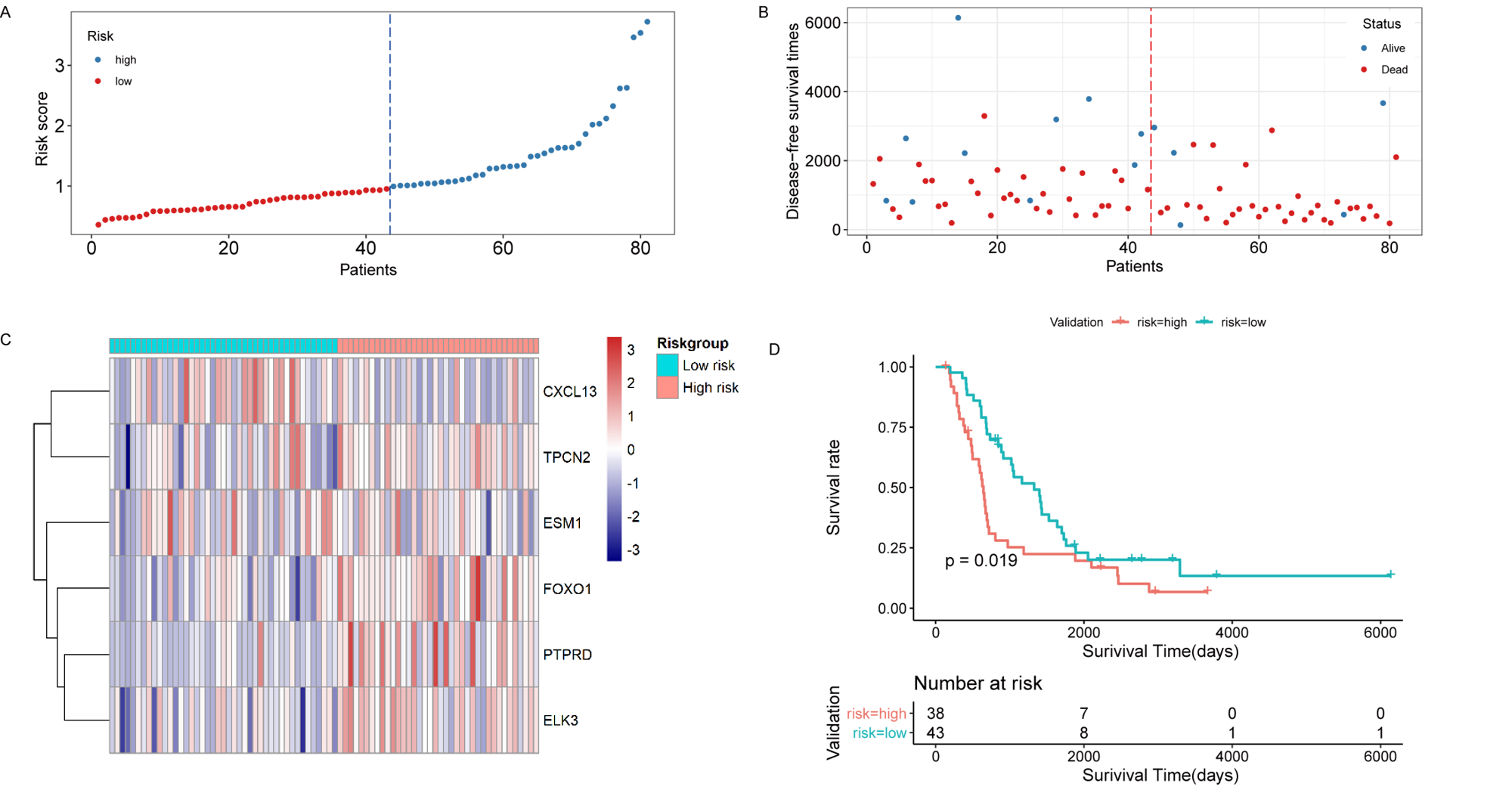

Supplement: Supplementary file 2 [file Image_2.tif]

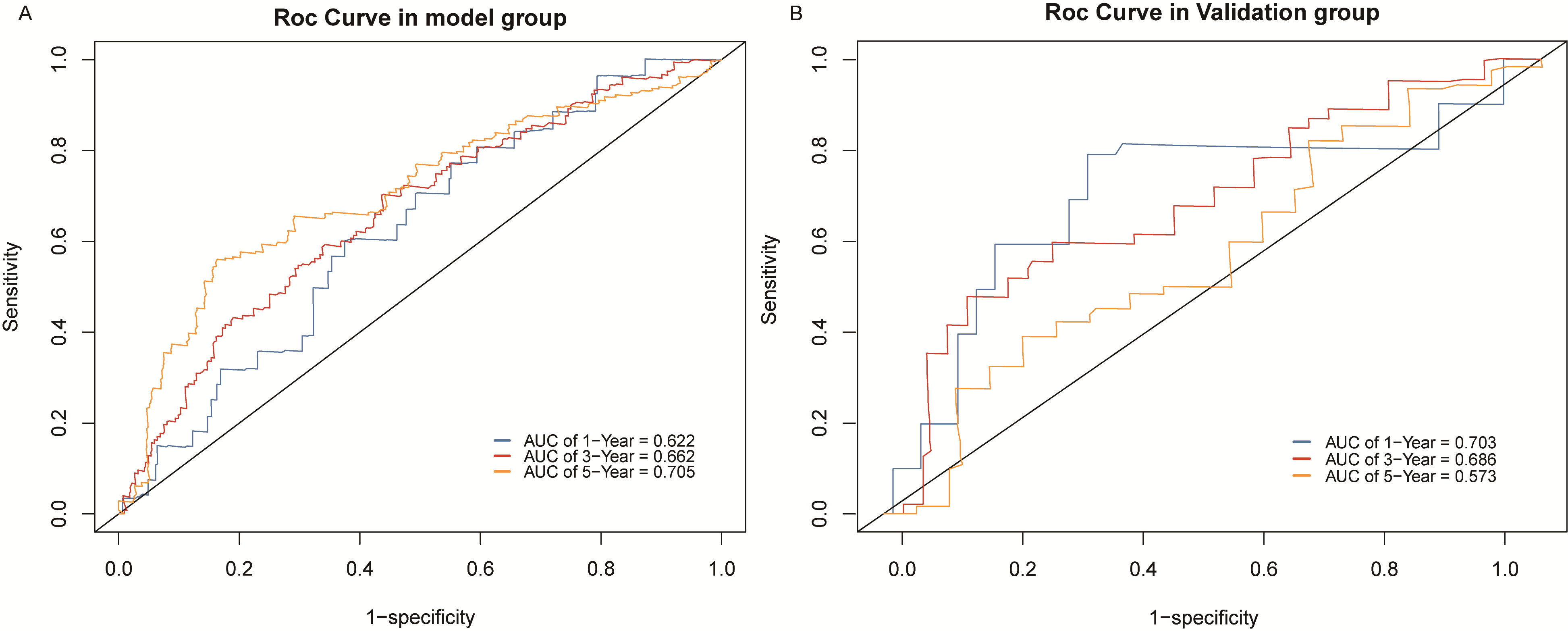

Supplement: Supplementary file 3 [file Image_3.png]

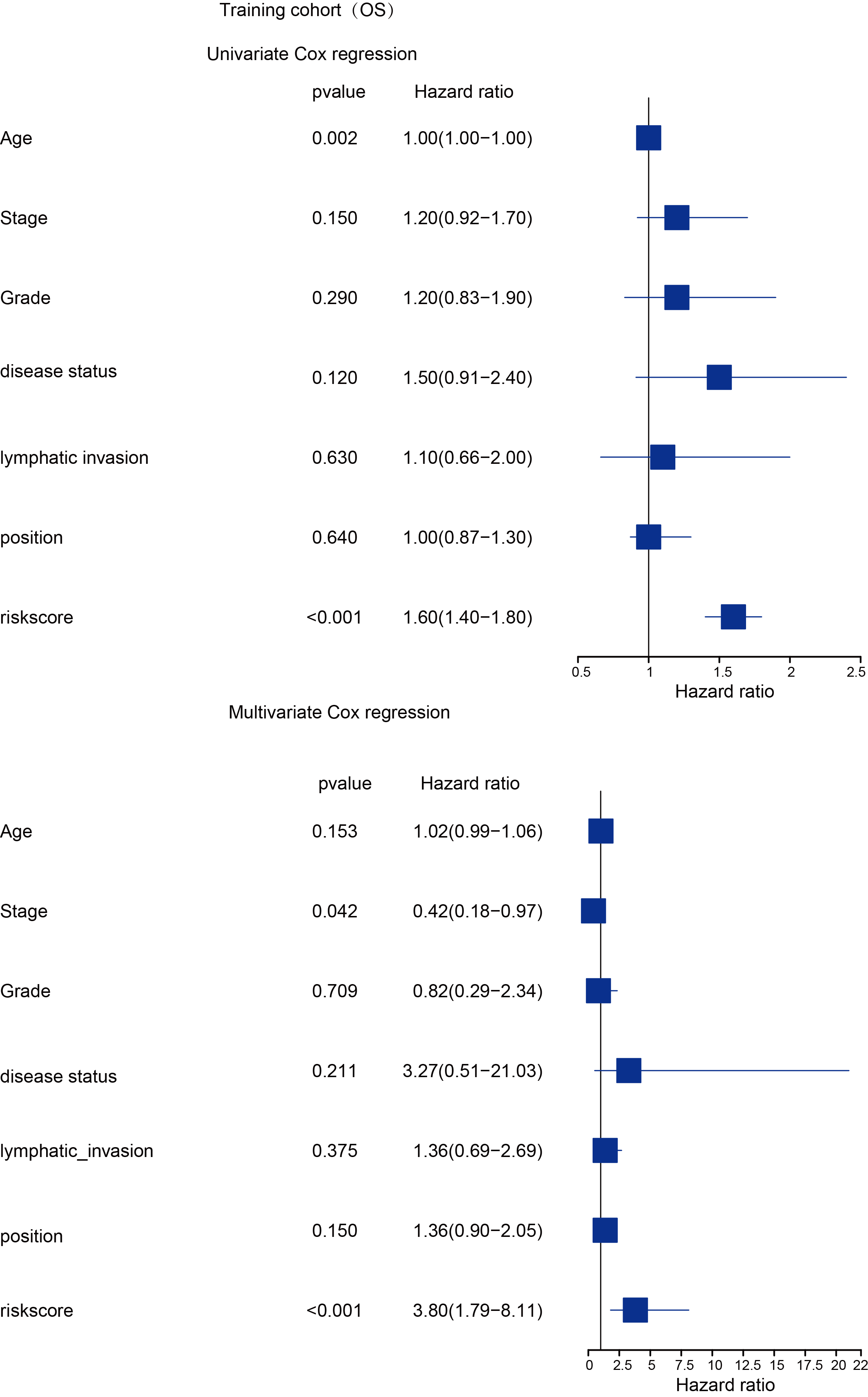

Supplement: Supplementary file 4 [file Image_4.png]

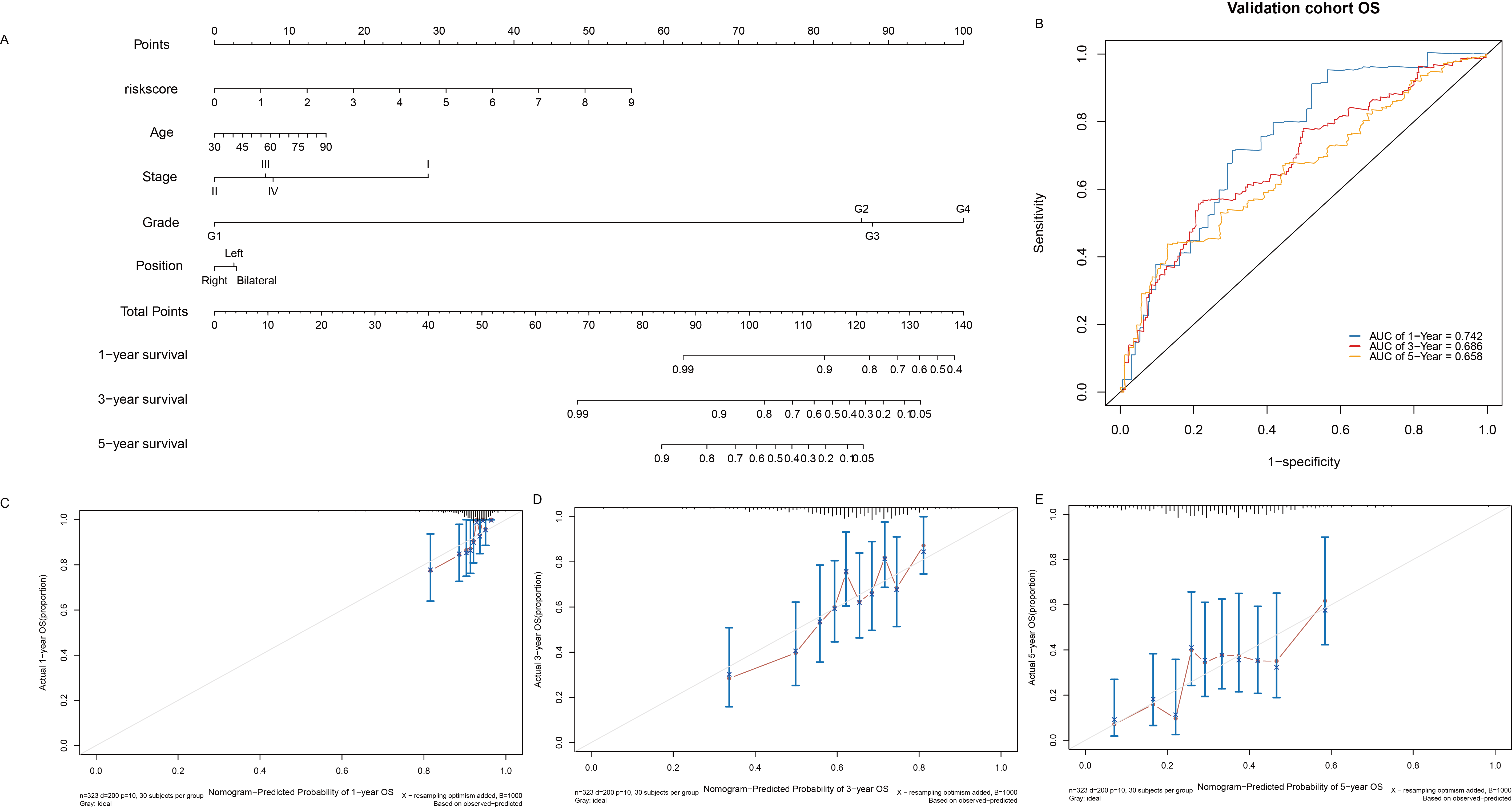

Supplement: Supplementary file 5 [file Image_5.png]

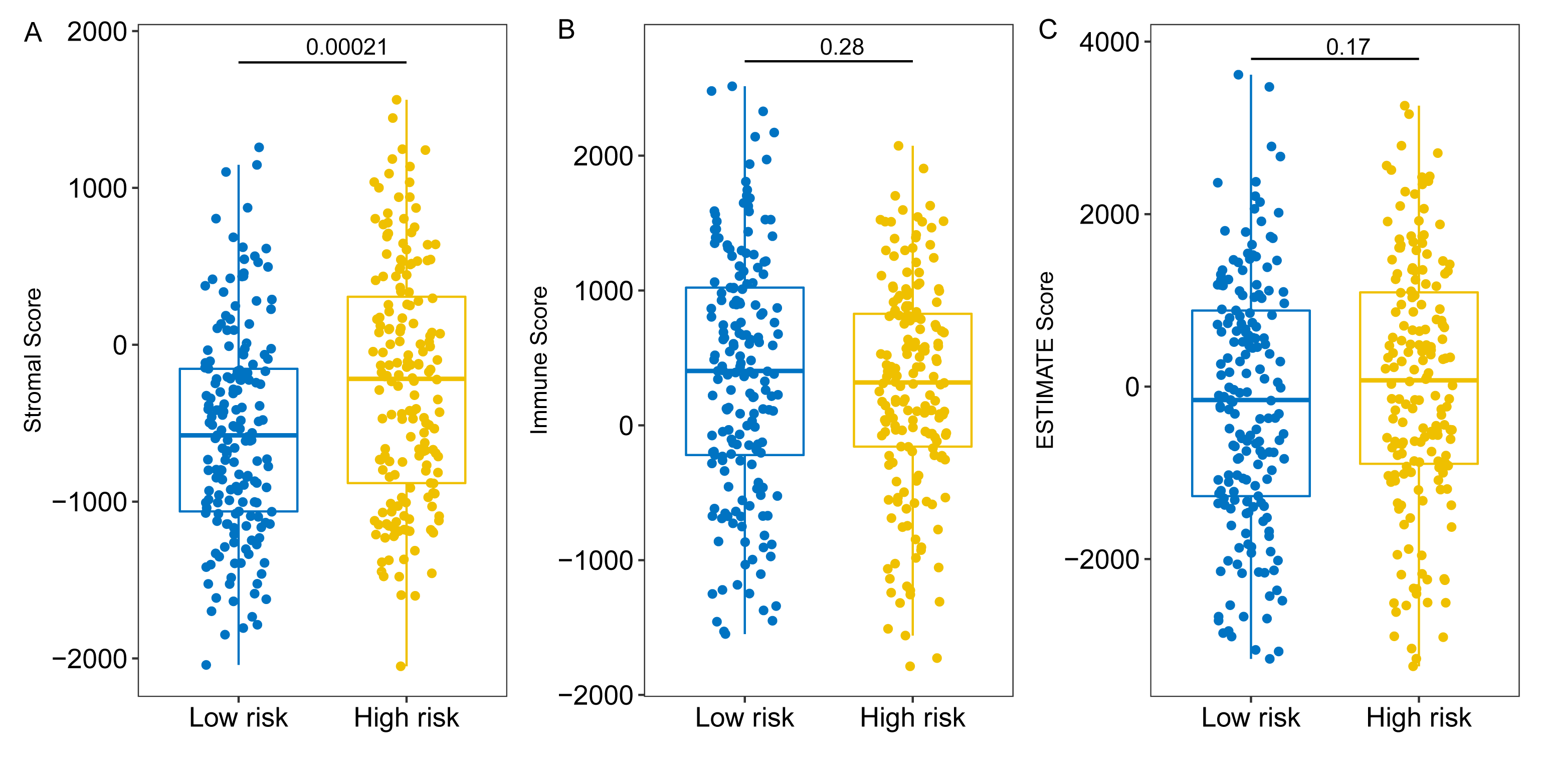

Supplement: Supplementary file 6 [file Image_6.png]
